# The many meanings of gross photosynthesis and their implication for photosynthesis research from leaf to globe

**DOI:** 10.1111/pce.12569

**Published:** 2015-06-25

**Authors:** Georg Wohlfahrt, Lianhong Gu

**Affiliations:** ^1^ Institute of EcologyUniversity of Innsbruck6020InnsbruckAustria; ^2^ European Academy of Bolzano39100BolzanoItaly; ^3^ Environmental Sciences Division and Climate Change Science InstituteOak Ridge National LaboratoryOak RidgeTN37831USA

## Abstract

(1)  Gross photosynthesis is a key term in plant biology and carbon cycle science, however has been used with different meanings by different communities
(2)  We review the history of this term and associated concepts to clarify the terminology and make recommendations about a consistent use of terms in accordance with photosynthetic theory.
(3)  We show that a widely used eddy covariance CO2 flux partitioning approach yields estimates which are quantitatively closer to the definition of true photosynthesis despite aiming at estimating apparent photosynthesis.

## Introduction

Photosynthesis is a complicated process and its research has a long history (Govindjee & Gest 2002). During this history, components of the photosynthetic process and their interconnections were unravelled only gradually. As the understanding of photosynthesis deepened, terminologies and definitions of key concepts often had to be revised, in many cases, repeatedly, in order to correct earlier mistakes and/or accommodate new findings. For example, the definition of ‘photosynthesis’ itself has been changed many times. American Scientist Charles Barnes (1858–1910) coined the word ‘photosynthesis’, although he preferred to use the word ‘photosyntax’ to describe the light‐driven reduction of CO_2_ to sugars in plants (Gest 2002). Earlier definitions of photosynthesis included simultaneous reduction of CO_2_ and evolution of O_2_, only to be corrected later after photosynthetic bacteria were discovered (Blankenship 2002). We are now facing a similar situation with the concept ‘gross photosynthesis’.

‘Gross photosynthesis’ is a term whose use has not been consistent in the long history of photosynthetic research. Historically, plant biochemists and physiologists, who studied photosynthesis at scales less than a leaf, did not consider photorespiration as part of photosynthesis even though photorespiration originates in chloroplasts (but photorespiratory CO_2_ is released via mitochondria into the cytosol), takes place simultaneously and competitively with CO_2_ reduction, and occurs only in light. This is because the discovery of photorespiration was relatively late (Decker 1955) and understanding its fundamental difference from the so‐called dark respiration (also known as mitochondrial respiration) took even longer time (Bowes *et al*. 1971). Once photorespiration was discovered and its mechanism understood, researchers started to use the term ‘true photosynthesis’ to describe the total CO_2_ fixation (i.e. a measure of carboxylation or equivalently oxygen evolution in the Hill reaction or all electrons generated by photochemical reactions), not allowing for any loss of CO_2_ through dark‐ and photorespiration. Meanwhile, the term ‘apparent photosynthesis’ was used to describe the difference between true photosynthesis and photorespiration, excluding dark respiration (Hew *et al*. 1969). Sometime during the following years, the term ‘gross photosynthesis’, which had been used earlier by experimental researchers of aquatic photosynthesis (e.g. Pratt & Berkson 1959), started to appear more frequently in the literature of general plant physiology. In the context of early aquatic studies, gross photosynthesis referred to the difference in oxygen concentrations between the light and dark bottles (Gaarder & Gran 1927; Pratt & Berkson 1959). In hindsight, gross photosynthesis in these early aquatic studies was actually equivalent to apparent photosynthesis because photorespiration occurs in the light bottle, but not in the dark bottle. Despite this early history, gross photosynthesis was used by plant biochemists and physiologists after the discovery of photorespiration to represent true photosynthesis. This use is continued in modern times as can be seen in influential textbooks of terrestrial and aquatic photosynthesis (e.g. Schopfer & Brennike 2010). Accompanying this changed meaning of gross photosynthesis, the term ‘net photosynthesis’ appeared and was used to describe the difference between true (gross) photosynthesis and the total (dark‐ and photo‐)respiratory losses of CO_2_, that is, the difference between apparent photosynthesis and dark respiration.

Both gross photosynthesis and net photosynthesis are now key concepts at all scales of photosynthesis research from molecular to leaf to canopy to globe. Unfortunately, the history outlined previously has often been disregarded in the contemporary use of the term ‘gross photosynthesis’ and, to some extent, ‘net photosynthesis’ as well. Consequently, these two terms, particularly gross photosynthesis, have been used inconsistently across space and time scales. This situation is especially serious in ecologically oriented studies of photosynthesis, which have the ultimate goal of quantifying gross primary productivity (GPP). Sometimes, the same researcher may use these two terms for different meanings in the same paper (e.g. Porcar‐Castell *et al*. 2014). The imprecise or incorrect use of different photosynthetic concepts has caused tremendous confusion.

Because of the critical importance of photosynthesis to local, regional and global carbon cycles, it is essential to clear this confusion so that estimates of carbon budgets can be compared across space, time and methods. The present paper attempts to do so. We will appeal for respecting the historical developments of photosynthetic terminologies, which means the following:

- Gross photosynthesis is true photosynthesis (carboxylation).
- Net photosynthesis is true photosynthesis minus photorespiration and dark respiration.
- GPP is intended as an integration of apparent photosynthesis (true photosynthesis minus photorespiration), NOT gross (true) photosynthesis.

We will present rationales behind these appeals and discuss their implications for modelling and measuring photosynthesis at multiple scales. Because the eddy covariance (EC) approach has been playing a foundational role in carbon cycle research (Baldocchi 2008), we will examine the actual meanings of canopy‐scale photosynthetic estimates inferred from EC flux measurements.

## Leaf‐Scale Photosynthesis Terminology

We start with a mathematical description of leaf net photosynthesis (*P*_n_) of C_3_ plants based on the theory of von Caemmerer & Farquhar (1981):

(1) $$P_{n}=V_{c}-0.5V_{o}-R_{\text{day}}\text{​}.$$

Here, *V*_c_ and *V*_o_ represent the rates of carboxylation (true photosynthesis) and oxygenation by the enzyme ribulose bisphosphate carboxygenase/oxygenase (Rubisco), respectively, and *R*_day_ represents daytime leaf respiration other than photorespiration. All terms have a unit of moles of CO_2_ per unit leaf area and time. 0.5*V*_o_ is photorespiration (often denoted as *R*_pr_). In Eqn (1), we use *R*_day_ to differentiate it from leaf dark respiration in the night, *R*_dark_ (respiration of a dark‐adapted leaf). The relationship between *R*_day_ and leaf dark respiration in the night will be discussed later. Here, we employ a sign convention by which component fluxes, such as true photosynthesis (carboxylation rate) and respiration, which do not change sign, are always referred to with a positive sign, while for net fluxes, such as net photosynthesis or the net ecosystem production (NEP), a positive flux represents a net uptake by a leaf or an ecosystem from the atmosphere and a negative flux the reverse. Equation 1 may also be expressed as

(2) $$P_{n}=V_{c}\;\left(1-\frac{\Gamma \text{*}}{C_{i}}\right)-R_{\text{day}}\text{​},$$

where Γ* represents the CO_2_ compensation point in the absence of *R*_day_ (Pa) and *C*_i_ stands for the CO_2_ partial pressure (Pa) inside the intercellular airspace. For convenience, in this formulation, we have ignored, as in most literature of large‐scale photosynthesis, the crucial importance of mesophyll diffusion (Sun *et al*. 2014a, 2014b).

**Figure 1:**
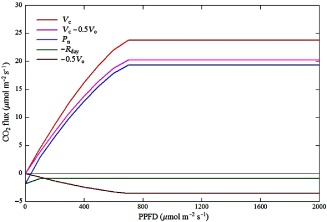
Graphical representation of the leaf photosynthesis terms by means of model simulations carried out with the Farquhar *et al*. (1980) model of net photosynthesis, using biochemical parameters from Wohlfahrt (2004). The apparent reduction of mitochondrial respiration in the light (maximum of 50% at high light) was accounted for according to Wohlfahrt *et al*. (2005). Leaf temperature (25 °C), intercellular CO_2_ (25Pa) and O_2_ (21 kPa) partial pressures were kept constant in all simulations.

Equations (1) and (2) state that leaf net photosynthesis is the result of the CO_2_ carboxylation minus CO_2_ losses through photorespiration (for every oxygenation of 1 mol of oxygen, 0.5 mol of CO_2_ is released) minus any (mostly) mitochondrial respiration. Both carboxylation and photorespiration occur only in light, whereas mitochondrial respiration continues in dark as well as in light. Equation (2) emphasizes the intrinsic and inseparable link between CO_2_ assimilation by carboxylation and release through photorespiration. The ratio Γ*/*C*_i_ represents the fraction (up to 50%; Schopfer & Brennike 2010) of carboxylated CO_2_ being released through photorespiration. Equations (1) and (2) hold at the scale of single leaves as well as plant canopies, provided appropriate spatial integration is applied (De Pury & Farquhar 1997). Figure 1 shows the various photosynthesis terms, based on simulations with the Farquhar *et al*. (1980) model, in a visual fashion.

## Photosynthetic Terms and the Central Role of Apparent Photosynthesis in Modelling and Measurements

Just to emphasize, with respect to the photosynthesis terminology, *V*_c_ corresponds to the term ‘true photosynthesis’, *V*_c_ − 0.5*V*_o_ to the term ‘apparent photosynthesis’ (Hew *et al*. 1969) and *V*_c_ − 0.5*V*_o_ − *R*_day_ to the term ‘net photosynthesis’. Unfortunately, this photosynthetic terminology has not been followed consistently. Both the true photosynthesis and the apparent photosynthesis have been referred to as gross photosynthesis (e.g. Schopfer & Brennike 2010; Porcar‐Castell *et al*. 2014), while net photosynthesis has often been used in place of ‘apparent photosynthesis’. Sometimes, this mixed use of net and apparent photosynthesis is intentional as in full sunlight, the day respiration of a healthy leaf is much smaller than its apparent photosynthesis. But, in other times, researchers may have not realized this term has been misused.

In the carbon cycle research community, a more confusing situation is caused by the use of gross photosynthesis interchangeably with gross primary production/productivity or gross ecosystem production/productivity, abbreviated as GPP or GEP, respectively. Such practice may be prompted by the shared appearance of the word ‘gross’ in these terms. However, this practice conflicts with the historical equivalence of gross photosynthesis with true photosynthesis. It also contradicts with what GPP is intended to be and with the way GPP is actually calculated. For carbon cycle research, knowing true photosynthesis is not as important as knowing apparent photosynthesis because photorespiration always accompanies and immediately reduces true photosynthesis. Consequently, it is more meaningful to calculate GPP as the spatial/temporal integration of apparent photosynthesis (i.e. true photosynthesis minus photorespiration), rather than true photosynthesis.

As far as we know, no carbon cycle researchers have calculated or intended to calculate GPP as an integration of true photosynthesis, even though they may use GPP and gross photosynthesis interchangeably. For example, Chapin *et al*. (2006) defined GPP as ‘the sum of gross carbon fixation by autotrophic carbon‐fixing tissues per unit area and time’. But from the context of that paper, it is clear that their gross carbon fixation was meant to be apparent photosynthesis, not true photosynthesis. GPP is also represented in most biogeochemical and land surface models as the difference between carboxylation rate (=true photosynthesis) and photorespiration (e.g. Bonan *et al*. 2011, Sun *et al*. 2014a). Interestingly, this calculation of GPP by modern modellers is consistent with the work of early pioneers of canopy photosynthesis research. In their now much celebrated study of canopy dry matter production, Monsi & Saeki (1953) used the following two equations for leaf and canopy, respectively:

(3) $$A=\frac{bI}{1+aI}-r,$$

(4) $$P=\frac{b}{Ka}\ln\left(\frac{1+aKI_{0}}{1+aKI_{0}e^{-KF}}\right)-rF.$$

According to the rather verbatim translation (from German to English) by Marcus Schortememeyer (Monsi & Saeki 2005), these two pioneers called *A* leaf net assimilation (equivalent to leaf net photosynthesis *P_n_* in Eqn (1)), *r* leaf respiration, and *P* canopy productivity (*a* and *b* are empirical constants, *K* extinction coefficient, *F* leaf area index, *I* light intensity inside canopy and *I*_0_ light intensity above canopy). They called *bI*/(1+*aI*) ‘leaf photosynthesis’. By analogy, Monsi and Saeki would have called the corresponding term in the canopy equation (the first term in the left side of Eqn (4)) ‘canopy photosynthesis’ (they however did not use this phrase explicitly). Although photorespiration does not appear directly in Monsi and Saeki's leaf photosynthesis and canopy photosynthesis, it should be considered as an integral part of both terms since *r* and *rF*, which are the only term left when *I*_0_ and therefore *I* are set to zero, clearly denote day respiration at leaf and canopy scales, respectively. Thus, Monsi and Saeki's leaf photosynthesis and canopy photosynthesis are apparent photosynthesis. The integration of apparent photosynthesis at the canopy scale over time leads to GPP.

True (gross) photosynthesis cannot be directly measured in natural conditions (Larcher 2001), although its approximation may be obtained under low oxygen concentrations (to supress photorespiration) or by feeding leaves with ^12^CO_2_ and ^13^CO_2_ in sequence and monitoring in‐ and out‐fluxes of these two labelled molecules (Haupt‐Herting *et al*. 2001). Apparent photosynthesis cannot be directly measured either but can be estimated in theory with differential approaches much like the light/dark bottle approach mentioned in the Introduction section. At the leaf scale, this can be achieved by measuring CO_2_ evolution in the dark and then adding this number to the net photosynthesis obtained in the light to get the apparent photosynthesis (but see the caveats discussed in the next section). The same principle can be and has been applied at the canopy scale. In the early 1900s, researchers already started to use translucent chambers to study the whole‐plant CO_2_ exchange (for a review, see Baldocchi & Amthor 2001). If a translucent chamber is covered with a dark cloth, the dark respiration of the whole plant would be measured. Measurements from the translucent chamber with and without the dark cloth could then be used to calculate the apparent photosynthesis of the whole plant.

Forests are too large to be encased in chambers, but the EC technique (Baldocchi 2008) offers several options, each with its own caveats. One option is to couple an overstory EC system with either an understory EC system or an automated soil chamber system (Baldocchi *et al*. 1987; Law *et al*. 1999; Misson *et al*. 2007). The sum of the net uptake measured by the overstory system and the flux by the understory system equals canopy true photosynthesis minus canopy photorespiration minus canopy day respiration minus stem respiration. This sum is the counterpart of leaf net photosynthesis (Eq. (1)) at the canopy scale and can be properly called canopy net photosynthesis (Baldocchi & Amthor 2001). To obtain canopy apparent photosynthesis, however, one will need estimates of canopy day respiration and stem respiration, as it is the sum of canopy net photosynthesis plus canopy day respiration plus stem respiration.

Stable carbon and oxygen isotopes have been used to partition net ecosystem exchanges of carbon dioxide measured by the EC approach into canopy photosynthesis and ecosystem respiration (Yakir & Wang 1996; Bowling *et al*. 2001; Griffis 2013). Furthermore, carbonyl sulfide (COS) (Campbell *et al*. 2008; Wohlfahrt *et al*. 2012; Berry *et al*. 2013) and sun‐induced chlorophyll fluorescence (Guanter *et al*. 2014; Parazoo *et al*. 2014) have been proposed as tracers of canopy photosynthesis. Detailed discussions of these approaches are beyond the scope of this short opinion article. However, we want to point out that it remains to be determined what terms of canopy photosynthesis can be resolved with their application. In the case of stable isotopes, uncertainties associated with fractionations by Rubisco, photorespiration and dark respiration and potentially large differences in isotope compositions among respirations from leaf, stem, root and microbes (e.g. Tcherkez *et al*. 2011; Ghashghaie & Badeck 2014) will complicate attempts to partition net fluxes into any components. For the COS approach, uncertain distributions of carbonic anhydrase, the enzyme that catalyses the assimilation of COS, within the mesophyll cell structures (Evans *et al*. 2009) and in the soil (Wingate *et al*. 2009) and potential emissions of COS from soil (Maseyk *et al*. 2014) will challenge COS as a tracer of different canopy photosynthetic components. Chlorophyll fluorescence is a signature of photosynthesis (Papageorgiou & Govindjee 2004). Because chlorophyll fluorescence intensity induced by sunlight is proportional to the electron transport rate from the photosystem II to photosystem I, which, in turn, is proportional to *V*_c_ − 0.5*V*_o_, it should be a measure of apparent photosynthesis. However, the relationship between chlorophyll fluorescence and apparent photosynthesis will be affected by temperature, radiation, water stress and other environmental variables and may not be linear.

Researchers have also exploited the fact that photosynthesis and respiration are driven by different environmental factors to infer the apparent photosynthesis from the EC measurements of the net exchange of CO_2_ between the atmosphere and the underlying ecosystem. This has been done in two ways. The first is that during nighttime, the net ecosystem CO_2_ exchange consists only of ecosystem respiration (=leaf dark + stem + root + microbial respirations), that is, ‘true photosynthesis’ and photorespiration do not occur (Reichstein *et al*. 2005). The other is that ecosystem respiration is not a direct function of photosynthetically active radiation (PAR), while both the true photosynthesis and the photorespiration are (Gu *et al*. 2002). Regardless of which way is exploited, both approaches can only estimate the apparent photosynthesis, not the true photosynthesis. Ironically, due to a potential difference between dark respiration in the night and dark respiration in the day (i.e. day respiration, which occurs in the light), the photosynthetic estimates from EC flux measurements with certain approaches may be closer to the stated but unintended gross (true) photosynthesis than to the unstated but intended apparent photosynthesis. This issue is addressed next.

## Implications for EC CO_2_ Flux Partitioning

EC CO_2_ flux measurements above active vegetation are characterized by fluxes of generally opposing sign during day and night. During nighttime, in the absence of photosynthetically active radiation, the nighttime net ecosystem production (NEP_n_) reflects only CO_2_ release to the atmosphere, that is,

(5) $${\text{NEP}}_{n}=-\left(R_{\text{dark}}+R_{\text{non-leaf}}\right)$$

where *R*_dark_ represents leaf respiration during darkness and *R*_non‐leaf_ collectively summarizes respiration from all other plant organs (e.g. wood, root) and heterotrophic organisms (microorganisms, animals). Here, we use the term NEP synonymously to net ecosystem CO_2_ exchange (NEE) and refer to Chapin *et al*. (2006) for a thorough discussion of the practical differences between the two terms, which are however neglected in the context of the present paper.

At the same temperature, *R*_dark_ is typically larger than *R*_day_ (0.2 < *R*_day_/*R*_dark_ < 1.3) (Heskel *et al*. 2013; Niinemets 2014) due to mitochondrial respiration being suppressed in the presence of light (Atkin *et al*. 1997) and/or because part of the CO_2_ produced by mitochondrial respiration is re‐fixed by photosynthesis (Pinelli & Loreto 2003). The degree to which *R*_day_/*R*_dark_ < 1 is however highly uncertain due to challenges in reliably estimating *R*_day_ (Gu & Sun 2014). In any case, due to the absence of radiation and thus photosynthesis, NEP_n_ has no contribution from photorespiration.

During daytime, *R*_day_ < *R*_dark_ (but see above) and NEP_d_, in addition to *R*_non‐leaf_, includes CO_2_ uptake by carboxylation and CO_2_ loss by photorespiration, that is,

(6) $${\text{NEP}}_{d}=V_{c}-0.5V_{o}-\left(R_{\text{day}}+R_{\text{non-leaf}}\right)$$

The partitioning algorithms (Falge *et al*. 2001; Reichstein *et al*. 2005; Lasslop *et al*. 2010b) that are presently used within the FLUXNET project (Baldocchi 2008) intend to solve Eqn (6) for the ‘apparent’ (*V*_c_ − 0.5*V*_o_) photosynthesis (even though they may state to estimate gross photosynthesis), which requires estimating *R*_day_ + *R*_non‐leaf_. To achieve so, the FLUXNET algorithms extrapolate the respiration terms in Eqn (5), which are collectively referred to as ecosystem respiration (*R*_eco_), to daytime conditions by parameterizing *R*_eco_ as a function of temperature (Reichstein *et al*. 2005; Lasslop *et al*. 2010b).

Because *R*_dark_ is typically, even though the actual degree is highly uncertain (Gu & Sun 2014), larger than *R*_day_, extrapolation of nighttime measurements to daytime conditions overestimates *R*_day_ and consequently the ‘apparent photosynthesis’. This issue has been known in the EC CO_2_ flux community for over a decade (e.g. Janssens *et al*. 2001) and Wohlfahrt *et al*. (2005) have shown this overestimation to amount to ca. 11–17%.

**Figure 2:**
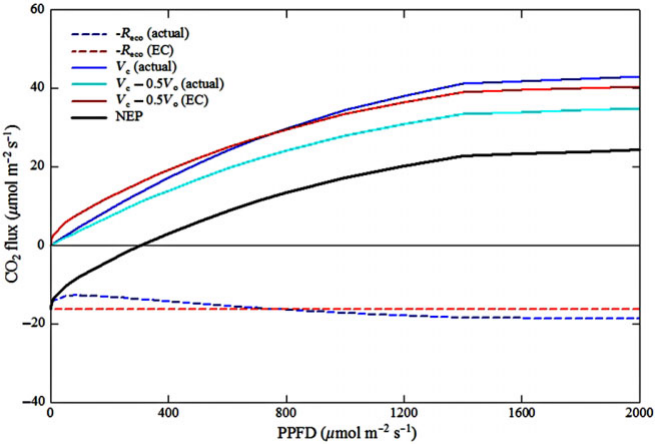
Simulation of ecosystem respiration (*R*_eco_), ‘apparent’ (*V*_c_ − 0.5*V*_o_) and ‘true’ (*V*_c_) photosynthesis using the canopy photosynthesis model by De Pury & Farquhar (1997), modified to include the apparent reduction (maximum of 50% at high light) of leaf mitochondrial respiration in the light according to Wohlfahrt *et al*. (2005) and a constant (equal to 40% of leaf dark respiration) source of non‐leaf respiration. This figure compares the actual ecosystem respiration, ‘apparent’ and ‘true’ photosynthesis with ecosystem respiration and the ‘apparent’ photosynthesis estimated through the flux partitioning approach. The leaf area index (2 m^2^ m^−2^), temperature (25 °C), intercellular CO_2_ and O_2_ partial pressures (25 Pa and 21 kPa), the fraction of diffuse radiation (0.1) and the sun's angle (65°) were kept constant in all simulations.

Because NEP_n_ overestimates *R*_day_ and does not include *R*_pr_, the FLUXNET estimate of apparent photosynthesis (GPP) may be quantitatively closer to the stated but unintended ‘true’ than the unstated but intended ‘apparent’ photosynthesis. To explore this possibility, we have used the sun/shade big‐leaf model by De Pury & Farquhar (1997), which consists of the Farquhar *et al*. (1980) model of leaf net photosynthesis integrated with a big‐leaf canopy radiative transfer model which distinguishes between sunlit and shaded leaf area fractions. The reduction of *R*_day_ compared with *R*_dark_ was included into the model of leaf net photosynthesis based on Wohlfahrt *et al*. (2005). Simulations were conducted by varying incident photosynthetically active radiation to generate light response curves of canopy scale *V*_c_, *V*_c_ − 0.5*V*_o_ and *R*_day_ (*R*_dark_ at zero light). The leaf area index (2 m^2^ m^−2^), temperature (25 °C), intercellular CO_2_ and O_2_ partial pressures (25 Pa and 21 kPa), the fraction of diffuse radiation (0.1) and the sun's angle (65°) were kept constant to this end. *R*_non‐leaf_ was parameterized as a fixed fraction (0.4) of *R*_dark_ for the sake of simplicity and the NEP_n_ and NEP_d_ were calculated according to Eqns (3) and (4), respectively. The FLUXNET partitioning approach was then mimicked by subtracting nighttime *R*_eco_, that is, *R*_dark_ + *R*_non‐leaf_ (Eqn (5)), from the calculated NEP_d_. As shown in Fig. 2, *R*_eco_ determined with the flux partitioning approach exceeded the true *R*_eco_ at low light levels because *R*_day_ was overestimated. The overestimation was larger than the value of *R*_pr_, which was relatively small at low light conditions. As a consequence, the estimated ‘apparent photosynthesis’ exceeded not only the correct ‘apparent photosynthesis’ (*V*_c_ − 0.5*V*_o_) but also the ‘true photosynthesis’ (*V*_c_). As the intensity of incident radiation increased, *R*_pr_ increased with the rising carboxylation rate and *R*_day_ decreased due to the progressive inhibition of R_dark_ and eventually *R*_eco_ estimated with the flux partitioning approach fell short of the true *R*_eco_ and the inferred ‘apparent photosynthesis’ settled between the correct ‘apparent’ and ‘true’ photosynthesis. Overall, the ‘apparent photosynthesis’ estimated with the flux partitioning approach produced estimates that were closer to the ‘true photosynthesis’ than the correct ‘apparent photosynthesis’.

**Figure 3:**
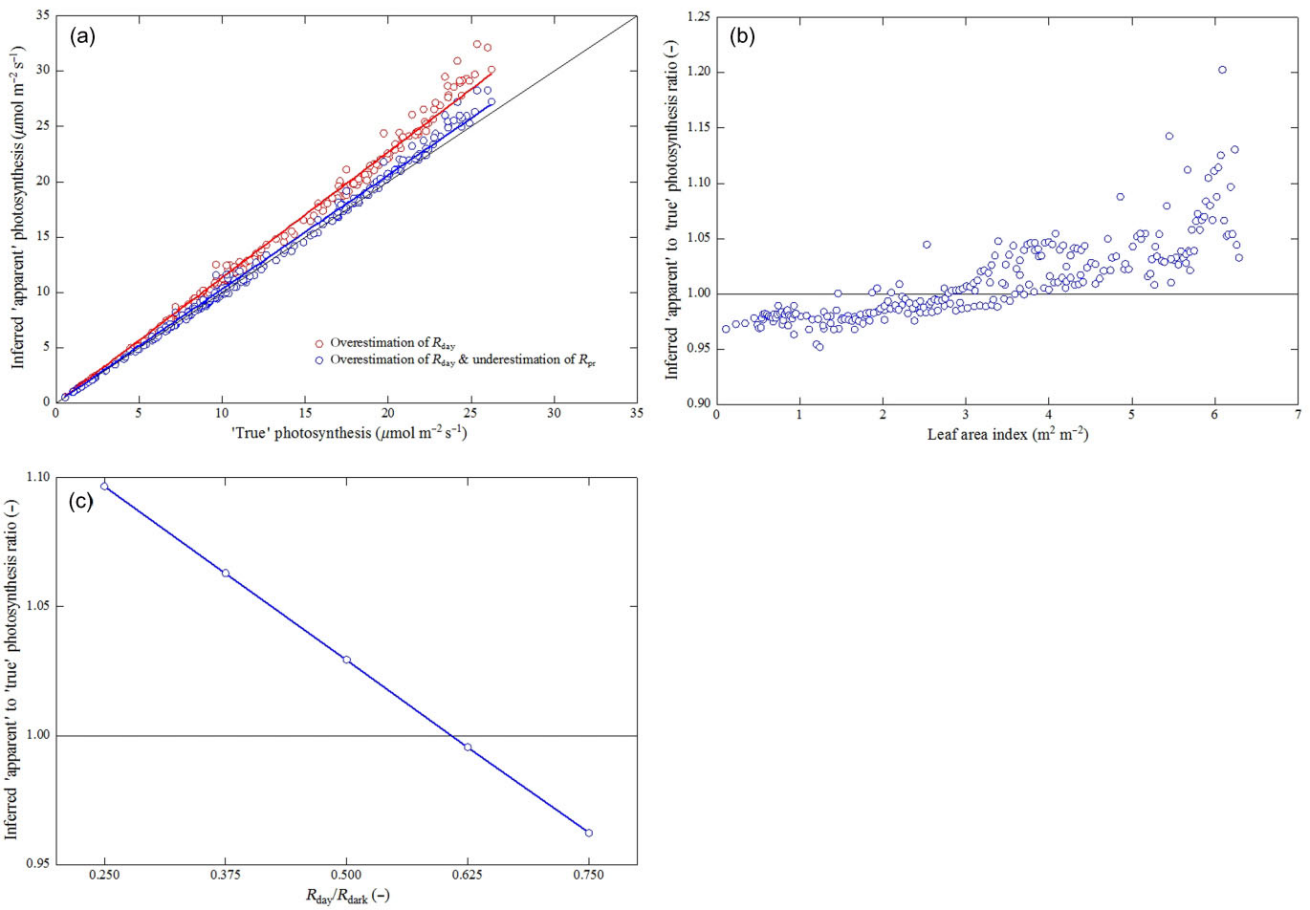
(a) Comparison between the ‘true photosynthesis’ (*V*_c_) and the ‘apparent photosynthesis’ inferred by means of the flux partitioning approach accounting only for the overestimation of leaf day respiration (*R*_day_; red symbols) and in addition for the underestimation of photorespiration (*R*_pr_; blue symbols). Solid lines represent linear regressions forced through the origin with slopes of 1.03 (blue line) and 1.13 (red line). (b) Ratio of inferred ‘apparent’ to ‘true’ photosynthesis (accounting for both the overestimation of leaf day respiration and the underestimation of photorespiration) as a function of the leaf area index as it varies during the course of the season. (c) Ratio of inferred ‘apparent’ to ‘true’ photosynthesis (accounting for both the overestimation of leaf day respiration and the underestimation of photorespiration) as a funtion of the ratio of *R*_day_/*R*_dark_. Simulations used measured half‐hourly seasonal values of air and soil temperature, direct and diffuse photosynthetically active radiation, leaf area index and soil respiration from a temperate mountain grassland from the study of Wohlfahrt *et al*. (2005). The intercellular CO_2_ and O_2_ partial pressures were kept constant throughout at 25Pa and 21 kPa, respectively.

The overestimation at low and the underestimation at high levels of incident radiation shown in Fig. 2 suggests that the ‘apparent photosynthesis’ estimated with the flux partitioning approach may converge to the ‘true photosynthesis’ over the daily cycle. In order to explore this issue, the model as described earlier was forced with seasonally varying measurements of environmental drivers (air and soil temperature, direct and diffuse photosynthetically active radiation) and leaf area index of a temperate mountain grassland based on the dataset used already by Wohlfahrt *et al*. (2005) for exploring the consequences of the reduction *R*_day_ compared to *R*_dark_ on the inferred apparent photosynthesis (GPP). *R*_non‐leaf_ was replaced with simulations of soil respiration as detailed in Wohlfahrt *et al*. (2005); otherwise, the same procedure as described earlier was followed. As shown in Fig. 3a, the daily average ‘apparent photosynthesis’ as determined with the flux partitioning approach overestimated the ‘true photosynthesis’ by only 3% (Fig. 3a; Wohlfahrt *et al*. 2005). Seasonal variations of the leaf area index turned out to be a major driver of the discrepancy between both approaches as the underestimation of the inferred ‘apparent photosynthesis’ with respect to the ‘true photosynthesis’ switched to overestimation at leaf area indices around 3 m^2^ m^−2^ (Fig. 3b). As noted already by Wohlfahrt *et al*. (2005), these simulations are highly sensitive to the degree of reduction of *R*_day_ with respect to *R*_dark_ and in addition to the ratio between *R*_dark_ and the maximum carboxylation efficiency (Heskel *et al*. 2013; Niinemets 2014). Understanding of both processes is presently, in particular compared to photorespiration, insufficient and thus their relative importance poorly constrained (Niinemets 2014). The sensitivity of the simulations to the ratio of *R*_day_/*R*_dark_ is exemplified in Fig. 3c, which shows how the ratio between the inferred ‘apparent photosynthesis’ and the ‘true photosynthesis’ changes as a function of *R*_day_/*R*_dark_. In our case study, a 10% in the *R*_day_/*R*_dark_ translated into a change in the ratio between the inferred ‘apparent photosynthesis’ and the ‘true photosynthesis’ of ca. 3% (Fig. 3c).

## Conclusions

We have reviewed the background of leaf photosynthesis and the associated terminology and showed that two differing definitions of gross photosynthesis are used in the literature. The first definition equates gross photosynthesis with the carboxylation rate, which historically has been referred to as ‘true photosynthesis’, while the second definition subsumes carboxylation with photorespiration, which historically has been referred to as ‘apparent photosynthesis’.

We further show that the commonly applied EC CO_2_ flux partitioning (Reichstein *et al*. 2005; Lasslop *et al*. 2010b) yields estimates of GPP which conceptually correspond to the definition of the ‘apparent photosynthesis’ due to the fact that the nighttime ecosystem respiration on which estimated daytime ecosystem respiration is based does not contain any information on photorespiration. The major new finding of this study is that despite being conceptually not compatible with the definition of ‘true photosynthesis’, GPP inferred by flux partitioning is quantitatively actually closer to the ‘true’ than the ‘apparent’ photosynthesis. This is due to an overestimation of daytime mitochondrial respiration with the flux partitioning approach. The actual degree of overestimation is shown to be the result of a complex interplay between biotic and abiotic influence factors and thus varies seasonally (Fig. 3b) and, although not tested here, very much likely between study sites. While GPP estimated with the flux partitioning approach was still somewhat overestimated in the investigated mountain grassland over the annual cycle, underestimation occurred during certain times as well (Fig. 3b) and might dominate under certain conditions.

A key uncertainty (Fig. 3c), and at the same time highly sensitive parameter (Wohlfahrt *et al*. 2005), is the actual degree to which leaf mitochondrial respiration is reduced in the light relative to darkness, which according to Niinemets (2014) and Heskel *et al*. (2013) varies between 20 and 130%. Due to technical challenges in reliably estimating *R*_day_, available *R*_day_/*R*_dark_ ratios should be viewed with caution and it is presently not clear whether this large degree of variation is reflective of biological variability or experimental artefacts. In addition, Pinelli & Loreto (2003) suggested that the reduction of *R*_day_ in the light might actually be an apparent one, the CO_2_ released by mitochondrial respiration being re‐fixed by photosynthesis. In the latter case or more generally if *R*_day_/*R*_dark_ ≈ 1, the flux partitioning approach would result in unbiased estimates of the ‘apparent photosynthesis’ (*V*_c_ − 0.5*V*_o_) and would underestimate the ‘true photosynthesis’ (*V*_c_) by the flux of photorespiration.

Finally, a word of caution is in order: The present study exclusively focused on issues of photosynthesis terminology in context with the approach of EC flux partitioning and did not quantify other uncertainties with this approach. The uncertainty of nighttime EC CO_2_ flux measurements is one of these issues (Aubinet 2008). Another one is that the extrapolation of nighttime CO_2_ flux measurements based on a simple temperature‐dependent model to daytime conditions ignores other drivers of diurnal variations in respiration rates, as it has been shown that temperature‐independent biotic and abiotic processes play a major role in modulating diurnal variations in ecosystem respiration components (e.g. Bahn *et al*. 2009; Vargas *et al*. 2011). Other authors (Vickers *et al*. 2009) have criticized that flux partitioning creates a spurious correlation between nighttime ecosystem respiration and apparent photosynthesis (but see reply by Lasslop *et al*. 2010a). Given the complexity of the processes involved and the associated theoretical and experimental uncertainties, it may be worthwhile to question how meaningful EC CO_2_ flux partitioning is and to seek other ways of exploiting the strong contrast between night and daytime net ecosystem CO_2_ exchange.

These measurement and methodological uncertainties call for strict use of photosynthetic terminologies so that communications among researchers and across disciplines can be facilitated. For all the purposes of what gross primary production (GPP) has been used for, this GPP has been and has to continue to be calculated as an integration of ‘apparent photosynthesis’ as knowing true photosynthesis without simultaneously knowing photorespiration is practically useless. Meanwhile, the carbon cycle research community should pay attention to the misuse of the concepts of gross photosynthesis and to some extent, net photosynthesis and stick to the historical use of these terms as outlined in the end of the Introduction section. To avoid confusion with GPP, we suggest that true photosynthesis is used in place of gross photosynthesis.
